# Modification of the Sweetness and Stability of Sweet-Tasting Protein Monellin by Gene Mutation and Protein Engineering

**DOI:** 10.1155/2016/3647173

**Published:** 2016-01-10

**Authors:** Qiulei Liu, Lei Li, Liu Yang, Tianming Liu, Chenggu Cai, Bo Liu

**Affiliations:** ^1^Department of Bioengineering, Qilu University of Technology, Jinan, Shandong 250353, China; ^2^Department of Food Science and Engineering, Qilu University of Technology, Jinan, Shandong 250353, China

## Abstract

Natural sweet protein monellin has a high sweetness and low calorie, suggesting its potential in food applications. However, due to its low heat and acid resistance, the application of monellin is limited. In this study, we show that the thermostability of monellin can be improved with no sweetness decrease by means of sequence, structure analysis, and site-directed mutagenesis. We analyzed residues located in the *α*-helix as well as an ionizable residue C41. Of the mutants investigated, the effects of E23A and C41A mutants were most remarkable. The former displayed significantly improved thermal stability, while its sweetness was not changed. The mutated protein was stable after 30 min incubation at 85°C. The latter showed increased sweetness and slight improvement of thermostability. Furthermore, we found that most mutants enhancing the thermostability of the protein were distributed at the two ends of *α*-helix. Molecular biophysics analysis revealed that the state of buried ionizable residues may account for the modulated properties of mutated proteins. Our results prove that the properties of sweet protein monellin can be modified by means of bioinformatics analysis, gene manipulation, and protein modification, highlighting the possibility of designing novel effective sweet proteins based on structure-function relationships.

## 1. Introduction

Monellin is a sweet protein extracted from the West African plant* Dioscoreophyllum cumminsii* [1]. Natural monellin consists of 94 amino acids with a molecular weight of 10.7 kDa. It consists of two chains A and B which are held together by noncovalent interactions. The A and B chains of the protein contain 44 and 50 amino acids, respectively [2, 3]. Monellin has a secondary structure consisting of five *β*-strands that form an antiparallel *β*-sheet and a 17-residue *α*-helix, as revealed in the resolved crystal structures of this protein and some of its variants (e.g., PDB: 1MOL, 2O9U) [4]. Monellin is proposed to be a promising sweetener. However, it is unstable at high temperatures or extremes of pH, which limits its extensive applications in food industry [5]. Single-chain monellin protein was created in which the two natural chains are joined via a Gly-Phe dipeptide linker, and site-directed mutagenesis has been extensively used to modify the functional properties of the protein [2]. Artificial single-chain monellin is as potently sweet as the wild type and is more stable upon temperature [2, 6–9]. However, few studies have reported the direct improvement of sweetness and thermostability of the protein by the gene mutation and protein modification techniques until now. Mutations of residues G1M, E2M, and E2N have been shown to result in an obvious improvement of the sweetness [10, 11]. Rega et al. reported a mutant Y65R with significant increase of sweetness and solubility in acidic conditions and compared the structure and function between the single-chain monellin and this mutant [12, 13]. Lee et al. studied the thermostability of various E23A variants with the circular dichroism analysis and succeed to transform these variants into tobacco chloroplasts [14]. However, detailed heat resistance and thermal denaturation as well as sensory evaluation of these mutants have not been investigated.

The sweet taste of monellin can only be detected by human and old-world monkeys in primates [15]. Until now, the mechanism by which the sweet proteins interact with and activate the sweet taste receptor-heterodimeric T1R2/T1R3 remains elusive. A wedge model suggests that sweet proteins interact with the large external cavity in the receptor and then induce the receptor activation [16]. We have reported that either T1R2 or T1R3 is responsible for the sweet taste difference towards monellin between human and squirrel monkey, and the electrostatic properties of the receptors probably mediate the species-dependent response to sweet-tasting proteins [17, 18].

Hydrophobic interactions, conformational entropy, and hydrogen bonding are believed to contribute most to monellin protein stability [19–23]. The functional analysis of wild type MNEI (single-chain monellin) and the G16A and V37A mutants showed an order of the thermal stability, WT (wild type)>G16A>V37A>G16A/V37A, and an order of sweetness threshold value, G16A>G16A/V37A>V37A>WT [24]. The lowest threshold value indicated the protein with most sweetness (the sweetest one). However, the least sweet mutant, G16A-MNEI, was not the least stable protein. This study indicates that there is no correlation between the stability and sweetness of the sweet protein monellin.

Another study pointed out that coulombic interactions are of primary importance for the function of monellin [25]. Charge-charge interactions play a less prominent role in protein assembly and stability compared to interactions involving hydrophobic core residues. However, it is suggested that the net charge of the protein surface can also affect the protein stability as well as its sweetness [26]. Furthermore, by investigating the behavior of single-chain monellin and a series of its variants, it was found that a reduced thermal stability is associated with an increased aggregation tendency [27].

Sweet protein monellin is approximately 3000 times sweeter than sugar [1]. However, the protein has a slow onset of sweetness and a lingering aftertaste. On the other hand, sweetness of monellin is pH and temperature dependent [28]. The protein is of taste at pH 2–9, and heat treatment over 50°C at low pHs denatures monellin protein with a loss of the sweetness [8]. Nevertheless, sweet proteins are extremely expensive, and they have about billions of dollars in global sales market per year. In this study, we aimed to increase the thermal stability and quality of sweetness of the sweet-tasting protein by site-directed gene mutagenesis and protein modification. Mutants were obtained by alanine substitution of amino acids at different sites in monellin protein, and their changes of thermal stability and sweetness threshold were evaluated. The results proved that the thermal stability and sweetness of the natural monellin protein can be improved by gene mutation technique, which could realize the industrial production of monellin and provide a new kind of sugar substitute for human being.

## 2. Materials and Methods

### 2.1. Chemicals, Enzymes, Bacterial Strains, and Plasmid

*Escherichia coli* strains DH5*α* and BL21-CodonPlus(DE3)-RIL and plasmid pET15b were from Novagen. Easy Pfu DNA polymerase and restriction enzyme FastDigest DpnI were from Beijing TransGen Biotech Co. and Thermo Scientific, respectively. All other molecular manipulation enzymes were from Takara Bio (Dalian, China). Ni Sepharose High Performance was from GE Healthcare. All other chemicals were of analytical grade and obtained from Sangon Biotech (Shanghai, China).

### 2.2. Cloning, Expression, and Purification of the Monellin Protein

According to the amino acid sequence of the single strand monellin sweet protein (GenBank: AFF58925.1), the monellin gene of full-length 294 bp was synthesized with the optimized codon usage by GenScript Co. The gene was then cloned into the plasmid pET15b with two restriction enzyme sites NdeI and BamHI at the N and C terminals, respectively. The recombinant plasmid was designated as pET15b-MNEI (single-chain monellin) and verified by DNA sequencing. The monellin variants were constructed on the basis of the recombinant plasmid. Primers of the MNEI mutants were designed to amplify the gene by PCR. The PCR product was digested by DpnI enzyme and then transformed and cultured in* E. coli* DH5*α* cells [17]. The plasmid of the mutated gene was purified and verified by DNA sequencing.

To overexpress the recombinant wild type and mutated proteins, the plasmids were transformed into* E. coli* BL21-CodonPlus(DE3)-RIL. An overnight culture in LB medium at 37°C was diluted 1 : 100 and grown until the OD_600_ reached 0.6 and then induced with 0.4 mM IPTG at 37°C for 4 h. Harvested cells were resuspended in lysis buffer (20 mM sodium phosphate buffer, 20 mM imidazole, and 500 mM NaCl, pH 7.4) and disrupted by 20 min sonication. The cell debris was removed by centrifugation at 10,000 rpm for 30 min. The proteins in the supernatants were purified by Ni Sepharose High Performance (nickel column affinity chromatography). The purified proteins were dialysed into MilliQ water and analyzed by SDS-PAGE.

### 2.3. Concentration Measurement and Sweetness Threshold Assay of the Wild Type and Variants Proteins

The concentration of proteins was measured by the Bradford method [29]. Double-blind taste assays were performed by a panel of ten healthy volunteer tasters, five males and five females, 20–60 years old [26]. The compounds tested were the MNEI protein, MNEI variants proteins, sucrose, and MilliQ water. Stock solutions of proteins were diluted by MilliQ water immediately prior to the taste assay. An initial series of protein samples with concentrations 0.25, 0.5, 0.75, 0.8, 0.9, 1.0, 1.5, 2.0, 2.5, 3.0, 5.0, 10, 15, 20, 45, 90, 100, and 150 *μ*g/mL was tested by one taster. To further determine the accurate threshold values of each sample, after the initial evaluation, we selected 0.05 *μ*g/mL as the concentration interval (0.25,0.3,0.35,0.4,0.45,0.5,…, 1 *μ*g/mL) for the proteins with sweetness threshold 0-1 *μ*g/mL and 0.1 *μ*g/mL as the concentration interval (1,1.1,1.2,1.3,1.4,1.5,…, 10 *μ*g/mL) for the proteins with sweetness threshold 1–10 *μ*g/mL, respectively. Samples were tasted in order starting from the lowest concentration until at least two consecutive concentrations were judged as sweet. Before each sample, tasters rinsed the mouth with tap water at least twice until no residual taste remained. Then, 1-2 mL of sample was taken into the mouth. The solution was held in the mouth for at least 10 s and then spit out. The tasters then graded the sample using the following notation to score their response (numbers indicate how these responses were scored): nonsweet and uncertainty at the threshold level of detection: 0; faintly sweet: 1.0; sweet: 2.0; very sweet: 3.0; intensely sweet: 4.0 [30]. Numerical scores for all tasters were combined to yield averages. The detection threshold was taken as the lowest concentration at which the taster recognized the sweetness as perceptible.

### 2.4. Thermostability Assay of the Sweet-Tasting Protein Monellin and Its Variants

100 *μ*L stock solutions of the dialyzed proteins were incubated in water bath at different temperatures for up to 10 h, respectively. A series of temperatures 40°C, 45°C, 50°C, 55°C, 60°C, 65°C, 70°C, 75°C, 80°C, 85°C, and 90°C were used and the proteins were taken at various times (2 h once). The proteins with water bath treatment were centrifuged to remove the sediment and the supernatants were analyzed by SDS-PAGE. Each experiment was carried out in three parallel experiments and the results were averaged.

### 2.5. Comparison of the His-Tagged and No-Tagged MNEI and Its Mutants

To investigate the probable effect of His-tag sequence on the properties of the sweet protein, we used the enzyme thrombin to cleavage and remove the His-tag sequence of the MNEI and its mutants E23A and C41A. Briefly, the overexpressed proteins in the supernatants obtained as described above were loaded on the nickel column (Ni Sepharose High Performance). The column was washed with distilled water, binding buffer (50 mM Tris-Hcl, 20 mM imidazole, and 150 mM NaCl, pH 7.4), and washing buffer (50 mM Tris-Hcl, 40 mM imidazole, and 150 mM NaCl, pH 7.4). Subsequently, the bottom of the column was plugged with a plug, followed by addition of 10 mL cleavage buffer (50 mM Tris-Hcl, 150 mM NaCl, and 2.5 mM CaCl_2_, pH 7.4) containing thrombin (100 U/mL, 0.5 mL). After incubation at 25°C for 16 h, the plug was removed and the no-tagged proteins were collected. This procedure ensured that the residual His-tagged proteins in the thrombin digested reaction mixture were removed. The resultant no-tagged proteins were then purified with an anion exchange column to remove thrombin. The sweetness threshold and thermostability of His-tag removed MNEI and its mutants were investigated with the same experimental procedures described above as the His-tagged proteins.

## 3. Results

**Figure 1:**
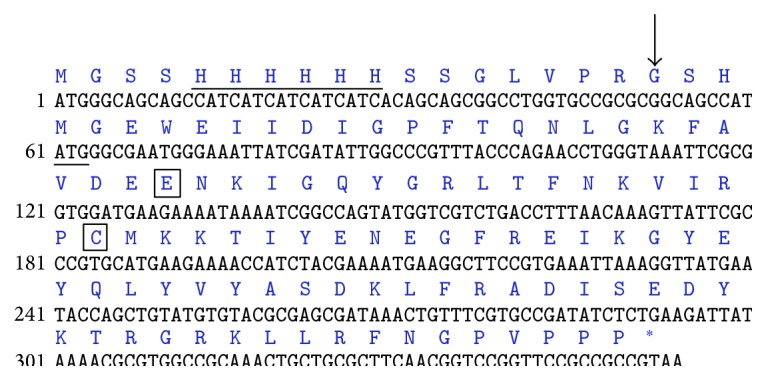
Nucleotide and amino acid sequence of single-chain monellin (MNEI) construct. The six histidines introduced at the N-terminal of the MNEI construct and the start codon are underlined, respectively. The thrombin cleavage site is denoted by an arrow. The stop codon of MNEI is annotated as asterisk. The E23A and C41A mutants sites with considerably improved thermostability or sweetness are boxed.

We analyzed the sequence and crystal structure of monellin protein (PDB: 2O9U). The amino acid and nucleotide sequences of single-chain monellin construct are presented in Figure 1. According to the published results, there is no specific domain, motif, or structural region which shows an obvious correlation with the sweetness or thermostability of the protein. A mutant G16A located at the *α*-helix of the protein has been reported to be crucial for its sweetness [24, 31]. Therefore, we focused on gene mutations in the amino acids of the *α*-helix, and the amino acids sites 10, 11, 12, 14, 15, 17, 18, 20, 21, 22, 23, 24, and 26 were selected and replaced by alanine, respectively (namely, P10A, F11A, T12A, N14A, L15A, K17A, F18A, V20A, D21A, E22A, E23A, N24A, and I26A), and the A19 was mutated to glutamate (A19E). Furthermore, C41 residue located in the second *β*-strand has been described to mediate the folding process of the protein, thus being selected for further mutagenesis analysis. The recombinant plasmids of MNEI and mutants were analyzed by DNA gel and sequencing technology, indicating that the single-chain monellin and its mutants were successfully constructed.

**Figure 2:**
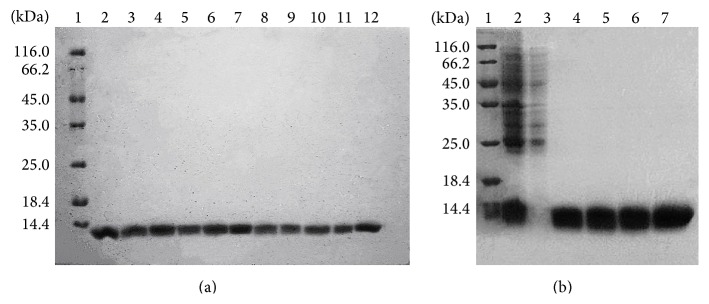
SDS-PAGE analysis of the purified MNEI and its variants. (a) 1: molecular weight marker; 2: purified MNEI (single-chain monellin) protein; 3: P10A; 4: F11A; 5: T12A; 6: N14A; 7: L15A; 8: K17A; 9: F18A; 10: V20A; 11: D21A; 12: E22A. (b) 1: molecular weight marker; 2: crude cell extract of A19E; 3: the supernatant of crude cell extract of A19E; 4: E23A; 5: N24A; 6: I26A; 7: C41A.

The overexpressed single-chain monellin and mutated proteins were analyzed by SDS-PAGE as shown in Figure 2. These results showed that the single-chain monellin and its mutants were successfully expressed and purified. We calculated the molecular mass of the recombinant monellin protein with the online bioinformatics software server (http://web.expasy.org/compute_pi/). The His-tag sequence was added to the amino acid sequence of monellin, and the total molecular weight of the recombinant protein was about 13 kDa.

A panel of ten healthy volunteers evaluated the sweetness of sugar, MNEI, and 15 variants as described in Section 2 [12, 27]. Protein solutions diluted over the range 0.25 to 150 *μ*g/mL were tasted starting with the most dilute, with water rinses between all samples. The concentration at which the sweet taste was first registered represented the sweetness threshold. The average threshold values for the proteins studied are presented in Table 1.

The results above indicated that six variants (N14A, F18A, D21A, E23A, N24A, and I26A) were consistent with the wild type single-chain monellin protein in sweetness. Notably, the sweetness threshold of C41A was lower than that of the wild type MNEI, demonstrating an improved sweetness of this mutant. The sweetness thresholds of other mutants were all increased, and the K17A mutant was the highest. Indeed, the K17A mutant tasted almost no sweet, and its thermostability was obviously reduced. It is worth noting that the A19E mutant was not successfully expressed, suggesting that this alanine residue plays a vital role in the correct folding of the protein.

**Figure 3:**
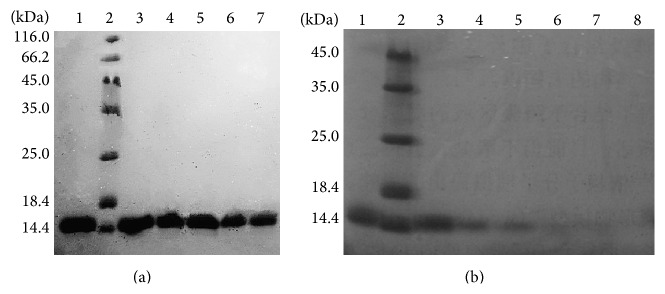
SDS-PAGE analysis of the thermostability of mutant E23A protein. (a) 1: E23A protein without heat treatment; 2: molecular weight marker; 3: heat treatment at 80°C for 2 h; 4: heat treatment at 80°C for 4 h; 5: heat treatment at 80°C for 6 h; 6: heat treatment at 80°C for 8 h; 7: heat treatment at 80°C for 10 h; (b) 1: E23A protein without heat treatment; 2: molecular weight marker; 3: heat treatment at 85°C for 30 min; 4: heat treatment at 85°C for 1 h; 5: heat treatment at 85°C for 2 h; 6: heat treatment at 85°C for 4 h; 7: heat treatment at 85°C for 6 h; 8: heat treatment at 85°C for 8 h.

Monellin and its variants were treated with the water bath experiment at different temperatures as described in Section 2. The results showed the maximum heat tolerance temperature of the different mutant proteins (Table 1). Most mutants displayed the remaining thermostability as the wild type MNEI. Of notice, the E23A mutant showed remarkable improvement of thermostability that the protein was stable and had no detectable change after incubation at 80°C for 10 h, and it was totally denatured after heat treatment at 85°C for 2 h (Figure 3). Moreover, the mutants P10A, F11A, N14A, N24A, and I26A resulted in slight increase of thermostability (about 5°C). These amino acids are located in the two ends of the *α*-helix, and their distribution is symmetrical. Our results conclude that manipulation of gene expression and modification of the original protein are effective for improvement and optimization of the properties of sweet-tasting proteins.

We also investigated the possible impact of His-tag sequence on the wild type MNEI and its mutants E23A and C41A. We used thrombin to cleavage and remove the N-terminal His-tag of the protein and conducted the same experimental procedures of sweetness threshold and thermostability for these no-tagged proteins as the tagged protein. We found that there was no difference of the sweetness threshold and thermostability between the tagged and no-tagged proteins (same values were obtained as shown in Table 1 for MNEI, E23A, and C41A), indicating that the His-tag sequence has no influence on the native protein.

## 4. Discussion

The relationship between the structure and function of sweet-tasting proteins has been an interesting subject for decades. Although many mutants of the sweet protein monellin have been investigated and the probable mechanisms for the sweetness have been proposed, intrinsic nature determining their sweetness and stability is still obscure. In this study, we revealed that the variants of E23A and C41A could lead to considerable improvement of the thermostability or sweetness of the sweet-tasting protein monellin, respectively. Several sites positively affecting the stability of the protein are symmetrically distributed at the two ends of *α*-helix region. These findings could provide meaningful guidance for molecular design and protein engineering of sweet-tasting proteins.

**Figure 4:**
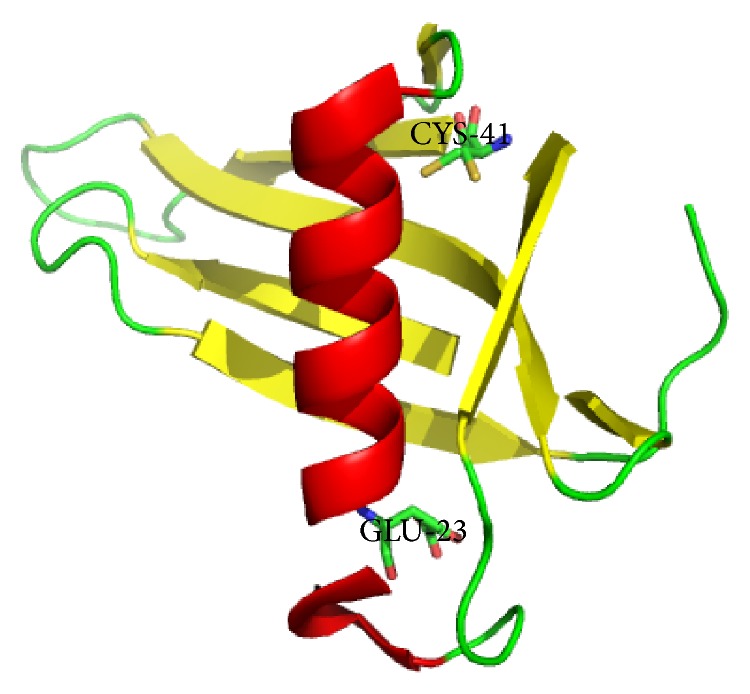
The structure of monellin derived from X-ray diffraction at a 1.15 Å resolution (PDB: 2O9U). The *α*-helix, *β*-sheet, and *β*-loop are colored in red, yellow, and green, respectively. The two residues with improved thermostability or sweetness in this study are labeled, rendered as sticks, and colored as atomic types.

E23 residue is located at the C-terminal end of the sole *α*-helix (Figure 4). Most of its total surface area (>95%) is buried in the core of the protein. This residue is highly ionizable in the native state of the folding protein, as evidenced by the modulation of the pH dependent stability of wild type and its mutant to alanine [32]. Our results are consistent with the previous finding that the maximum heat resistance temperature of the mutant E23A is improved to 85°C relative to that of the wild type MNEI (55°C). The enhancement of mutated protein should be due to the rationalization that replacement of this unpartnered ionizable residue from the hydrophobic core of the protein can stabilize the native state of monellin. Other mutants located in both ends of the *α*-helix (P10A, F11A, N14A, N24A, and I26A) could only slightly improve the thermostability. These residues are probably involved in the allosteric regulation or the hydrogen bonds network affecting the structural and functional properties of monellin. Nevertheless, the A19E mutant led to a complete insolubility, indicating that this residue may play a critical role in the proper folding of the native protein.

C41 residue is located at the second *β*-strand and is also buried, with its thermostability being slightly improved (Figure 4). This residue plays a concerted role with E23 to account for the pH dependent stability of the protein at pH > 8 [32]. The sweetness of mutant C41A is obviously increased. Another mutant K17A located at the center region of *α*-helix significantly reduced the sweetness of the protein. It appears that residues mediating the sweetness of monellin are scattered on the three-dimensional structure of the protein (e.g., G1, E2, G16, R39, and C41), but no unified mechanism could account for the sweet origin of sweet-tasting proteins [10, 11, 24]. It is interesting to further investigate the synergetic or additive effect of these residues for the sweetness of protein.

Because of the low stability at the heat and acid conditions, the extensive applications of monellin in food industry are unpractical now. Other similar sweet proteins such as thaumatin (3000 times sweeter than sucrose), brazzein (2000 times), mabinlin (375 times), curculin (550 times, induction by citric acid or Vc can produce a strong sweet), and miraculin (which has no sweet taste, but it can make citric acid sweet) have been used in research or industry [16, 28]. For example, thaumatin has been used in food, beverage, and chewing gum. In this study, we hope to find highly sweet and thermostable monellin mutants through the gene mutation experiments. We found that the stability of E23A mutant protein was the most prominent, with the highest heat resistance temperature reaching 85°C. Furthermore, the C41A mutant showed a lower sweetness threshold and increased sweetness than that of the wild type protein. These two mutants thus could be promising sweet substitutes for potential food applications. Taken together, our results confirm that the properties of sweet-tasting proteins can be modified based on the structure-guided molecular design.

## 5. Conclusions

Through the study of the thermal stability and the sweet threshold of the mutants of single-chain monellin (MNEI), we found that two mutants C41A and E23A exhibited improved sweetness or thermal stability. Our results highlight that manipulation of gene expression and modification of the original protein are effective for improvement and optimization of the properties of sweet-tasting proteins.

## Figures and Tables

**Table 1:** Sweetness threshold and maximum heat resistance temperature of monellin and its variants.

Name	Sweetness threshold (*μ*g/mL)	Maximum heat resistance temperature (°C)
Sucrose	10000 ± 150	—

MNEI	0.8 ± 0.05	65
P10A	3.0 ± 0.4	70
F11A	2.0 ± 0.25	70
T12A	10 ± 1.1	55
N14A	0.9 ± 0.1	70
L15A	2.0 ± 0.2	45 ± 5
K17A	>450	<40
F18A	0.9 ± 0.05	55 (50)
A19E (insoluble)	—	—
V20A	2.5 ± 0.5	45
D21A	1.0 ± 0.2	65 (45)
E22A	2.0 ± 0.3	60
E23A	0.8 ± 0.1	85
N24A	0.9 ± 0.15	75 (70)
I26A	0.8 ± 0.05	70 ± 5
C41A	0.5 ± 0.05	70

The protein thermostability was expressed as the maximum heat resistance temperature at which the protein displayed completely denaturation. The temperature in brackets indicated that protein was only partially denatured at this temperature. —: undetected.
